# Selective Doping in Silicon Carbide Power Devices

**DOI:** 10.3390/ma14143923

**Published:** 2021-07-14

**Authors:** Fabrizio Roccaforte, Patrick Fiorenza, Marilena Vivona, Giuseppe Greco, Filippo Giannazzo

**Affiliations:** Consiglio Nazionale delle Ricerche, Istituto per la Microelettronica e Microsistemi (CNR-IMM), Strada VIII, n. 5—Zona Industriale, 95121 Catania, Italy; patrick.fiorenza@imm.cnr.it (P.F.); marilena.vivona@imm.cnr.it (M.V.); giuseppe.greco@imm.cnr.it (G.G.); filippo.giannazzo@imm.cnr.it (F.G.)

**Keywords:** silicon carbide, 4H-SiC, ion implantation, selective doping, electrical activation, post-implantation annealing, power devices, JBS, MOSFET

## Abstract

Silicon carbide (SiC) is the most mature wide band-gap semiconductor and is currently employed for the fabrication of high-efficiency power electronic devices, such as diodes and transistors. In this context, selective doping is one of the key processes needed for the fabrication of these devices. This paper concisely reviews the main selective doping techniques for SiC power devices technology. In particular, due to the low diffusivity of the main impurities in SiC, ion implantation is the method of choice to achieve selective doping of the material. Hence, most of this work is dedicated to illustrating the main features of n-type and p-type ion-implantation doping of SiC and discussing the related issues. As an example, one of the main features of implantation doping is the need for post-implantation annealing processes at high temperatures (above 1500 °C) for electrical activation, thus having a notable morphological and structural impact on the material and, hence, on some device parameters. In this respect, some specific examples elucidating the relevant implications on devices’ performances are reported in the paper. Finally, a short overview of recently developed non-conventional doping and annealing techniques is also provided, although these techniques are still far from being applied in large-scale devices’ manufacturing.

## 1. Introduction

Silicon carbide (SiC) is a semiconducting material that possesses excellent physical and electronic properties, making it the best choice for the new generation of high-power and high-temperature electronic devices [1]. In fact, its wide band gap, high critical electric field and high thermal conductivity enable the fabrication of devices with superior performances with respect to the silicon ones, in terms of breakdown voltage, specific on-resistance and maximum operation temperature [2]. In particular, owing to its larger band gap (3.2 eV) and isotropic mobility along the a- and c-axis of the crystal, the hexagonal 4H-SiC is the most widely used polytype, for power electronics application [3]. As a matter of fact, enormous progress has been recorded in 4H-SiC power devices technology over the last three decades [4,5,6].

The first technological milestone was the introduction of the SiC Schottky diode into the market by Infineon in 2001, which was followed by a rapid market growth, leading to the development of a family of diodes with a voltage rating ranging from 600 V to 3 kV [5]. Then, the release of the first SiC MOSFET by CREE in 2011 definitively accelerated the adoption of SiC devices in many applications requiring a high energy efficiency, e.g., electric vehicles, photovoltaic inverters, industrial motors, etc.

During this thirty-year-long history of SiC, ion implantation of the material has been widely investigated, essentially because it represents the method of choice to achieve selective doping in power devices. In this context, many comprehensive regular and review papers have discussed the most relevant implications of ion implantation of SiC, e.g., crystal damage, amorphization and recrystallization, doping, annealing, contact formation, etc. [7,8,9,10,11,12,13,14,15,16].

While ion implantation is now a consolidated method for selective doping of SiC devices, other non-conventional doping techniques have also been explored by the scientific community in the last decade, including alternative annealing techniques, laser activation processes in specific environments, high-temperature annealing of metal films based on p-type dopant species, etc. [16].

The present paper aims to provide a concise review of selective doping techniques in SiC power devices. Clearly, great attention will be paid to the ion-implantation doping. In particular, after an introduction on the main fundamental issues related to implantation doping and electrical activation in SiC, some case studies on n-type and p-type doping in SiC devices technology will be presented, discussing the implications on the devices’ performances. Finally, a short overview of recently developed non-conventional doping and annealing techniques will be provided.

## 2. Background on Selective Doping in SiC Power Devices

Controlling the n-type and p-type doping of SiC is possible in a wide range, thus being a big advantage in terms of devices’ fabrication with respect to other wide band-gap semiconductors. In particular, nitrogen (N) or phosphorus (P) are used for n-type doping, while aluminum (Al) is employed as a p-type dopant of the material. Although boron (B) has also been initially proposed as a p-type dopant for SiC, today, it is not used as a dopant species because of its large ionization energy (∼350 meV) [17]. These dopant species become electrically active when they are incorporated in substitutional position in the SiC lattice. In particular, nitrogen is substitutional at the C sites, while phosphorus, aluminum and boron are substitutional at the Si sites.

The ionization energies of the dopant species depend on the occupied lattice site, i.e., whether the site is hexagonal or cubic. As an example, the ionization energy of nitrogen and phosphorous donors is relatively small and, hence, their ionization ratio is already reasonably high (from 50% to nearly 100%) at room temperature. On the other hand, the ionization energy of aluminum is much larger (200–250 meV), and for that reason, typically, incomplete ionization (5–30%) of the acceptors occurs at room temperature [18]. Table 1 summarizes the values of the ionization energies and the solubility limits of the main dopant species (nitrogen (N), phosphorous (P), aluminum (Al) and boron (B)) for the 4H-SiC polytype [1,3,4].

The experimental observations demonstrated that the ionization energy, *E_A_*, decreases with increasing the doping level, according to the relation proposed by Pearson et al. [19] and Efros et al. [20]:

(1) $$E_{A}=E_{0}-\alpha N^{\frac{1}{3}}$$

where *E*_0_ is the ionization energy in lightly doped materials, *N* is the doping level and *α* is a constant, which is typically in the range of (2–4) × 10^−5^ meV cm [1].

Silicon (Si) has dominated the semiconductor devices scene for several decades. In Si, both diffusion and ion implantation techniques are widely employed for selective doping during electronic devices’ fabrication [21]. Clearly, selective doping by a diffusion process is less invasive than ion implantation, where energetic ions introduce a variety of points and extended defects in the crystal lattice. However, ion implantation can enable a better control of the doping profile by multiple energies and doses of the implanted species.

**Figure 1:**
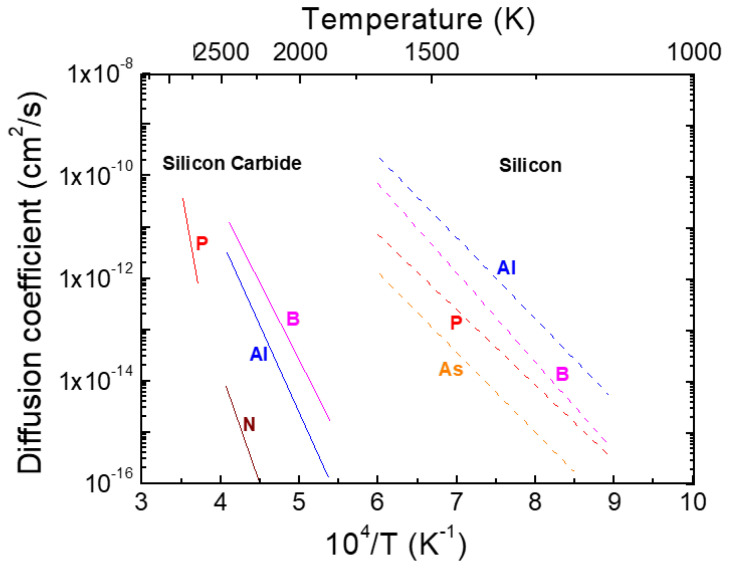
Arrhenius dependence of the diffusion coefficients of different dopant species in silicon carbide (continuous lines) and silicon (dashed lines). The data are taken from [1,13,22,23,24,25].

One of the peculiarities of silicon carbide is that due to the high Si-C bond strength, the diffusion coefficients of dopant species in the material are extremely low, even at very high temperatures (above 1500 °C). Figure 1 reports an Arrhenius plot of the diffusion coefficients for the major dopants in SiC and Si [1,13,22,23,24,25]. As can be seen, the diffusion coefficients of dopant impurities in SiC are in the order of 10^−17^–10^−15^ cm^2^ s^−1^ at about 1800 °C, i.e., several orders of magnitude lower than those of Si dopants (10^−14^–10^−13^ cm^2^ s^−1^) at 1000 °C. Therefore, since diffusion practically does not occur, ion implantation represents the method of choice to achieve selective doping in SiC devices.

**Figure 2:**
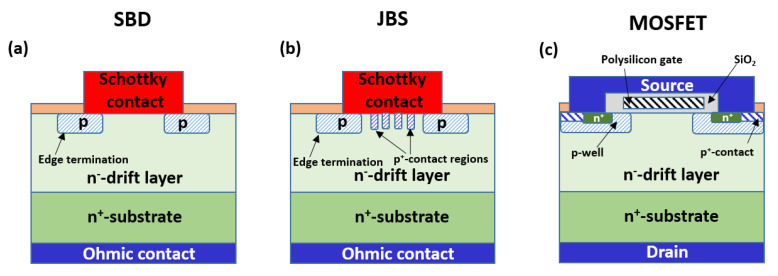
Schematics of SiC unipolar power devices: (**a**) Schottky Barrier Diode (SBD), (**b**) Junction Barrier Schottky (JBS) diode and (**c**) Metal Oxide Semiconductor Field Effect Transistor (MOSFET). Various selectively doped implanted regions (e.g., edge termination, p+-contact regions, p-well) are present in these devices.

Figure 2 shows the schematics of common SiC unipolar power devices: Schottky Barrier Diode (SBD), Junction Barrier Schottky (JBS) diode and Metal Oxide Semiconductor Field Effect Transistor (MOSFET).

As can be seen, in these devices, ion implantation is used to create selectively doped regions, e.g., source/drain regions, p^+^-contact regions, the guard ring and edge termination, the p-well, etc.

The typical doping levels and implantation depths of the main implanted junctions found in those SiC devices are summarized in Table 2.

Clearly, since the doping level of these regions critically influences the device performances, the control of the electrical activation of the dopant is a crucial issue in SiC device technology. In the next sections, the main issues in n-type and p-type doping of SiC by ion implantation will be discussed.

## 3. n-Type and p-Type Ion-Implantation Doping of SiC

As specified in Section 2, the most common dopant species in SiC are phosphorous and nitrogen for n-type, and aluminum for p-type doping, typically introduced by ion implantation in the crystalline matrix. Clearly, the greatest disadvantage of this technique is the generation of damage in the crystal structure that, for high-dose implantation, can also lead to the amorphization of the material [26,27,28].

In general, implantation at room temperature (RT) results in a high density of defects. Hence, hot implantation, i.e., ion-implantation doping at high temperature (400–1000 °C), is carried out to dynamically annihilate the defects created by the ion cascade [28,29].

Moreover, post-implantation annealing steps (typically at temperatures above 1500 °C) are also needed in order to electrically activate the dopant species, bringing them into substitutional lattice sites, and to further reduce the implantation-induced damage [28].

**Figure 3:**
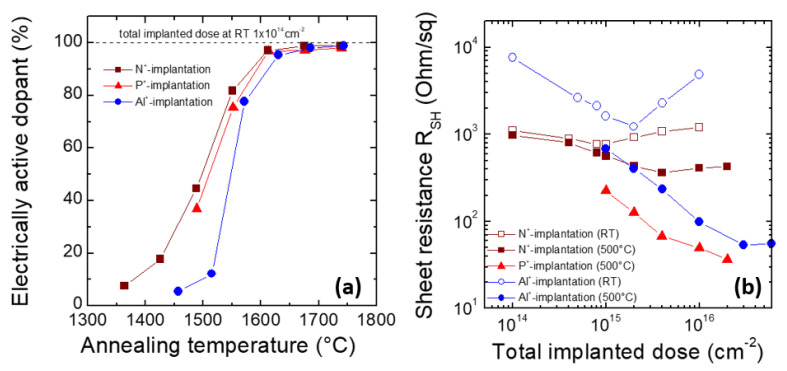
(**a**) Electrically active dopant percentage in N^+^-, P^+^- and Al^+^-implanted SiC. The implantation was performed at room temperature with a total dose of 1 × 10^14^ cm^−2^. (**b**) Sheet resistance as a function of the implanted dose in N^+^-, P^+^- and Al^+^-implanted SiC layers. The implantations were performed either at room temperature or at 500 °C. Post-implantation annealing was carried out for 30 min in Ar at 1700 °C (N and P) and 1800 °C (Al). The data are taken from [1,30,31].

Figure 3a shows the electrically active fraction of both n-type and p-type dopant species (N, P and Al), implanted at room temperature in SiC, with an implantation dose of 1 × 10^14^ cm^−2^, as reported in the studies of Kimoto et al. [1,30]. In particular, implantations at different energies (50–720 keV) and doses have been carried out to obtain a 400 nm thick “box-like” profile, with a concentration of 2 × 10^18^ cm^−3^. The electrically active fraction of the dopant is defined as the ratio between the donors (or acceptors) concentration (e.g., measured by C-V analyses) and the total implanted atoms (e.g., determined by SIMS). As can be seen in Figure 3a, the electrically active fraction for n-type and p-type dopants is still very low after annealing temperatures of 1400–1500 °C. However, with increasing the annealing temperature (1500–1600 °C), a significant increase of the activation is observed, until an almost complete activation is reached at 1650 °C. At this implanted dose, no significant differences in the electrical behavior of the implanted layer are observed if the implantation is carried out at elevated temperatures, i.e., up to 800 °C. However, by increasing the implantation dose, the electrical activation of the dopant species becomes more difficult, due to the generation of lattice damage, and “hot implantation” becomes mandatory.

As an example, Figure 3b depicts the sheet resistance of 4H-SiC implanted either at room temperature or at 500 °C, as a function of N-, P- and Al-implanted dose [31]. Ion implantations were carried out at different energies and doses, in order to obtain a “box-like” profile, with a thickness of about 200 nm. In these samples, the post-implantation annealing was performed for 30 min in Ar, at 1700 and 1800 °C, for n-type and p-type dopants, respectively. As can be observed, while for implanted doses lower than 1 × 10^15^ cm^−2^ there is no significant difference in the activation of room temperature or hot implantation, for high implantation doses, a significant improvement of the electrical properties of the layers is achieved by carrying out the implants at 500 °C.

As can be observed in Figure 3b, for the n-type doping, a better activation is achieved by P^+^- with respect to N^+^-implantation. A similar observation was made by Capano et al. [32], who compared the sheet resistance of 4H-SiC layers implanted at 600 °C with either P or N at a high dose and annealed at 1700 °C. In particular, they argued that the formation of N_2_ molecules or other nitrogen-based complexes in SiC are responsible for the lower activation ratio of nitrogen with respect to phosphorous. Laube et al. [33] observed a similar electrical activation of P- and N-donors for implanted concentrations up to about 2–5 × 10^19^ cm^−3^. Such a critical concentration represents an upper limit for electrically active N-donors, while P-donors can be activated at concentrations above 10^20^ cm^3^, also due to their higher solubility limit in SiC (see Table 1).

Based on the above results, today, n-type doping in 4H-SiC MOSFETs is typically obtained using P^+^-implantation. In addition, for devices’ fabrication, the implantation temperature is generally limited at 500 °C, since at higher temperatures the formation of extended defects may occur, which cannot be recovered by post-implantation annealing [34].

In order to increase or optimize the electrical activation of the implanted species in SiC, co-implantation of the common n-type and p-type dopants with either C or Si has been proposed by Troffer et al. [17]. In fact, theoretically, the co-implantation of C atoms can favor the electrical activation of Al and B acceptors in SiC, since an excess of C interstitials increases the probability for Al or B atoms to occupy Si sublattice sites, where they can act as shallow acceptors [17]. As an example, B in 4H-SiC acts as a shallow acceptor when it is in the substitutional Si atoms position. Hence, the co-implantation with C or Si atoms can be used to control the activation of B acceptors. In fact, C co-implantation has been seen to improve the electrical activation of B acceptors in 4H-SiC by increasing the free hole concentration, while Si co-implantation decreases the electrical activation [35].

Zhu et al. [36] monitored the effect of n-type P co-implantation with C- or Si-atoms. In particular, while C co-implantation resulted in a lower sheet resistance than Si co-implantation after high-temperature post-implantation annealing, no relevant improvement in the dopant electrical activation with respect to the P-implantation only was demonstrated [36].

Finally, in the case of Al and C co-implantation (20% of Al^-^ dose), Negoro et al. [31] observed an increase of the free hole concentration by a factor of 1.5–2.5 with respect to the sample implanted only with Al at the same dose. However, at the highest implanted Al concentration (>6 × 10^16^ cm^−2^), the beneficial effects of C co-implantation were very small, because of the generation of defects reducing the free hole concentration.

Clearly, the co-implantation technique is a more complex and expensive processing step than a conventional single-ion-implantation. In addition, the aforementioned literature results are often controversial. For those reasons, in spite of the potential benefits in terms of activation, this approach has not found application in real devices’ fabrication.

From the physical point of view, a limiting factor for the electrical activation of the implanted dopants is the formation of deep levels, acting as compensation centers, which occur upon irradiation and high-temperature annealing [37]. The compensating centers reduce the net-free carriers’ (electrons or holes) density in the implanted material, and this effect can be particularly pronounced in p-type-doped Al-implanted SiC layers [17,38,39,40,41]. Hence, the compensation must be taken into consideration when estimating the electrical active fraction of the implanted dopants in SiC by electrical measurements.

**Figure 4:**
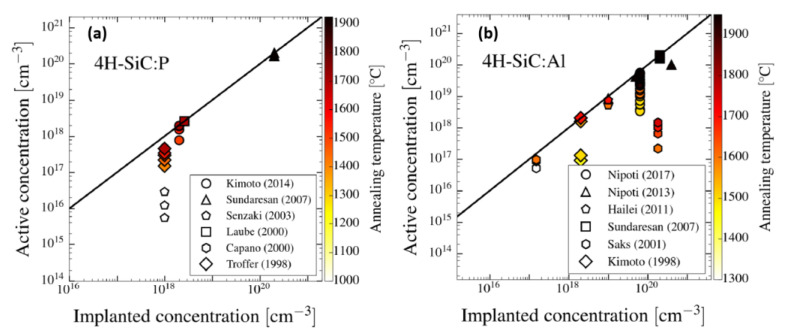
Survey of literature data on (**a**) P and (**b**) Al active concentration as a function of the implanted concentration at different post-implantation annealing temperatures. The straight lines indicate 100% electrical activation. The figures have been reproduced from [49] with permission from the author (A. Tofil).

A final practical consideration regards the post-implantation annealing time. In fact, most of the experimental works on electrical activation of implanted dopants in SiC refer to the “steady-state” activation, i.e., considering long annealing time, enabling the maximum electrical activation for that implanted concentration and annealing temperature. In general, ion-implanted species in SiC can be activated efficiently by furnace annealing above 1600 °C for an annealing time of around 30 min, and the electrical activation can be increased with increasing the post-implantation annealing temperature. This general trend is summarized in Figure 4, which reports some relevant literature findings on steady-state activation of P- and Al-implanted 4H-SiC at different annealing temperatures [1,30,33,42,43,44,45,46,47,48]. The straight lines in the figures indicate 100% activation. As can be seen, the deviation of the experimental data points from the 100% activation line is higher for the case of p-type Al-doping, due to the more significant compensation effect.

Nipoti et al. [50] presented the transient behavior of electrical activation of the Al p-type dopant in 4H-SiC, investigating the effect of the annealing time. In particular, they have shown that for Al concentration <1 × 10^19^ cm^−3^, while the Al atoms placed in substitutional sites are almost constant with the annealing time, the resistivity of the implanted layer decreases and the holes’ density increases with increasing annealing time, mostly due to a decrease of the compensation [50].

In this context, Šimonka et al. [51] recently analyzed various time-dependent (transient) literature data on the electrical activation of P and Al in 4H-SiC. They observed that the time constants of the transients for activation are in the order of several hours for post-implantation annealing temperatures lower than 1400 °C, and decrease significantly (down to some tens of minutes) at higher temperatures (>1700 °C). Based on this model, Toifl et al. [52] carried out a detailed analysis of the effects of post-implantation annealing conditions on the electrical characteristics of 4H-SiC MOSFETs, predicting both acceptor and donor concentrations at different annealing times, temperatures and total implanted concentration. These recent results provide a useful method for device manufacturers/designers to predict the dependence of the MOSFETs characteristics on the post-implantation annealing conditions.

## 4. Surface Roughness of SiC Implanted Layers

Beyond implantation-induced defects in the bulk material, another critical issue typical of SiC is represented by the surface degradation caused by the high-temperature annealing. In fact, for annealing temperatures above 1000 °C in vacuum, Si desorption from the SiC surface starts to occur, leading to surface graphitization [1]. Moreover, an increase of the surface roughness associated with the formation of “step bunching” occurs upon high-temperature post-implantation annealing. In particular, it has been observed that the surface roughness of implanted SiC increases with the annealing time and temperatures above 1500 °C [53,54].

The first attempt to mitigate the effect of Si desorption from 4H-SiC surface was presented by Capano et al. [53,55], who demonstrated an improvement of the surface roughness by carrying out the post-implantation annealing process in a silane (SiH_4_) overpressure or by placing some Si pieces in the annealing chamber.

Later, a similar approach to control the surface morphology of Al-implanted 4H-SiC layers was reported in a systematic investigation by Rambach et al. [56]. In this case, during post-implantation annealing at 1700 °C, the Si overpressure was achieved either by adding SiH_4_ or by placing the sample in a SiC-coated crucible.

**Figure 5:**
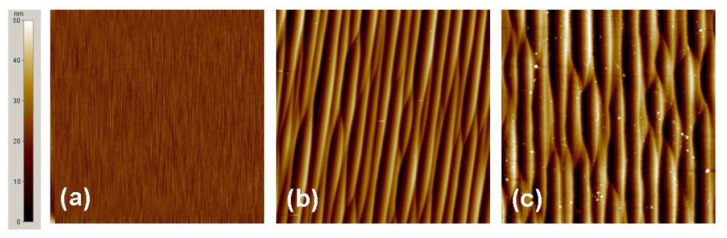
Surface morphology of 4H-SiC samples acquired by 10 × 10 μm AFM scans: (**a**) non-implanted sample, RMS = 0.12 nm; (**b**) n-type P-implanted sample, RMS = 7.89 nm; (**c**) p-type Al-implanted sample, RMS = 10.9 nm, after 1650 °C/30 min annealing. Adapted with permission from [57]. Copyright 2009 © Trans Tech Publications, Ltd.

The effects of P- and Al-implantation and high-temperature annealing on the surface morphology of 4H-SiC were also studied by Weng et al. [57] by means of atomic force microscopy (AFM), and are illustrated in Figure 5.

In particular, Figure 5a shows the surface morphology of a non-implanted 4H-SiC sample. As can be seen, small parallel steps, due to misorientation angle of the crystal, are visible on the sample surface. The root mean square (RMS) roughness of the sample remains practically unchanged even after multiple energy levels (30–150 keV) of P- and Al-implantation at temperatures of 300–400 °C and doses in the order of 10^15^ cm^−2^. However, after annealing at 1650 °C for 30 min, in Ar + SiH_4_ ambient conditions, both the P- and Al-implanted layers exhibit a similar morphology, consisting of large steps parallel to the original small ones observed before implantation (Figure 5b,c).

These results clearly indicate that the step bunching is associated with the ion implantation-induced damage in the surface region, where a high density of broken bonds is created, making the surface atoms highly mobile during high-temperature annealing. Moreover, they demonstrate that Si overpressure cannot completely solve the surface roughness issue.

The use of a capping layer during high-temperature annealing can be more efficient to reduce the step bunching and, hence, the surface roughness. In particular, today, the carbon (C)-capping layer is the most widely diffused approach to prevent Si desorption and step bunching formation. The C-capping layer is typically formed by an annealing of a standard photoresist at temperatures of about 800 °C [58,59,60]. In some cases, sputtering of a carbon layer on the sample surface is used instead of photoresist. Then, after the post-implantation annealing process, the C-capping layer is removed by an oxidizing process, e.g., a thermal oxidation at 800–900 °C, or by plasma treatments.

**Figure 6:**
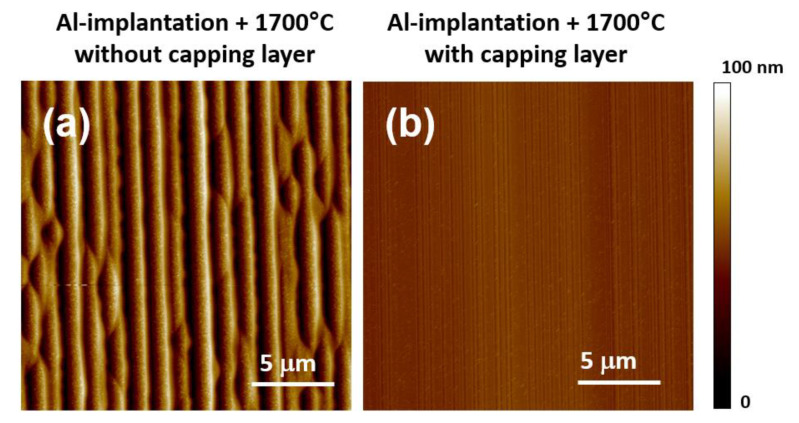
Surface morphology of high-dose Al-implanted 4H-SiC samples after annealing at 1700 °C (**a**) without capping layer (RMS = 19 nm) and (**b**) with capping layer (RMS = 2.4 nm). Adapted with permission from [61]. Copyright © 2021 IOP Publishing Ltd.

As an example, Figure 6a, b show the surface morphology of a n-type 4H-SiC epitaxial sample implanted at different energies (30–80 keV) with a total high dose (1.3 × 10^15^ cm^−2^) of Al^+^-ions, and then annealed at 1700 °C for electrical activation, either with or without a protective carbon-capping layer formed by an annealed photoresist layer [61]. The AFM scan in Figure 6b was acquired after capping layer removal with an oxidation process. As can be seen, while the sample subjected to high-temperature post-implantation annealing without the capping layer shows a pronounced surface roughness RMS (about 19 nm) with the presence of “step bunching” on the surface (Figure 6a), a smooth morphology (RMS = 2.4 nm) is preserved in the sample annealed with the capping layer (Figure 6b), comparable to the pristine material.

Obviously, the post-implantation annealing conditions and, consequently, the morphology and electronic properties of the implanted SiC surfaces, can have a strong influence on the electrical properties of the contacts and devices fabricated on them.

As an example, the surface roughness of Ti/Al Ohmic contacts on p-type implanted 4H-SiC, formed with an annealing at 950 °C, was significantly higher (43.5 nm) in the contacts fabricated on a rough SiC surface, with respect to that of contacts fabricated on p-type implanted layers processed with a carbon-capping layer (20.8 nm) [61]. Moreover, the Ti/Al contacts formed on the protected (smoother) SiC surface exhibited, on average, a lower specific contact resistance (in the order of 10^−4^ Ωcm^2^) with respect to those formed on the unprotected (rougher) SiC surface [61].

Additionally, the effects of post-implantation annealing conditions and surface roughness (determined by AFM) on the channel mobility of SiC MOSFETs have been discussed by different authors, who reported controversial results. In fact, Haney et al. [62] observed that the MOSFET channel mobility is not significantly affected by post-implantation annealing between 1200 and 1800 °C, by protecting the SiC surface with a carbon-capping layer during annealing. However, Naik et al. [63] reported that although the interface trap density was higher for the MOSFETs annealed without a capping layer, their channel mobility was higher compared to that of devices annealed without the graphite cap. Hence, they pointed out that an increased microscopic surface roughness induced by the capping layer was the limiting scattering mechanism, rather than the presence of interface traps. Interestingly, in the presence of a pronounced “step bunching” in the MOSFET channel annealed without any protection, an anisotropy of the drain current has been observed by Lee et al. [64]. More recently, Frazzetto et al. [65] measured a higher field effect channel mobility (40 cm^2^V^−1^s^−1^) in 4H-SiC lateral MOSFETs processed without a carbon-capping layer, with respect to that measured in the devices processed with a capping layer (24 cm^2^V^−1^s^−1^). All these results indicate the difficulty to correlate the surface roughness, determined by AFM, with the channel mobility. In this respect, Liu et al. [66] argued that the low mobility in the high-field region is limited by the sub-nanometer scale surface roughness. In general, based on the temperature dependence of the mobility, Coulomb scattering due to the presence of trapped charges at the SiO_2_/SiC interface has often been indicated as the main limiting factor for the mobility [65,67]. However, in the 4H-SiC MOSFET inversion channel, a large fraction of electrons are trapped at the interface states. Under these conditions, an increasing number of electrons can become mobile with increasing the temperature, thus leading to the experimentally observed positive temperature coefficient of the mobility. Therefore, the “electron trapping effect” is a more correct interpretation of the mobility behavior [4].

In conclusion, based on the tendency of implanted SiC surfaces to degrade upon severe thermal budgets, today, the use of a graphite carbon cap during post-implantation annealing is commonly adopted by device manufacturers to control the surface roughness at the critical interfaces in JBS and MOSFET based on 4H-SiC.

## 5. Effects of Selective Implantation Doping on Relevant SiC Devices Parameters

As illustrated in Section 2, selectively implanted n-type- and p-type-doped regions are key parts of both JBS and MOSFETs devices, and have a notable impact on the devices’ performances. In the following subsections, specific examples related to the MOSFET channel and to the contact regions of either MOSFETs or JBS are reported.

### 5.1. Doping Effects on Threshold Voltage and Channel Mobility of 4H-SiC MOSFET

As reported in Table 2, intermediate p-type Al-doping levels (in the order of 10^17^–10^18^ cm^−3^) are employed in the definition of the p-body region of 4H-SiC MOSFETs, where the inversion layer is formed below the interface with the gate oxide (see device schematic in Figure 2).

The doping concentration of the p-well has a strong impact on the SiC MOSFET parameters, such as the threshold voltage and the channel mobility [68]. Hence, the control of electrical activation of Al and the doping level of the p-well are fundamental to obtain a good reproducibility of the electrical devices’ characteristics.

In particular, the threshold voltage, *V*_th_, in a lateral MOSFET can be analytically expressed as [69]:

(2) $$V_{\mathrm{th}}=V_{\mathrm{FB}}+2\Phi_{F}-\frac{qN_{\mathrm{ox}}}{C_{\mathrm{ox}}}+\frac{1}{C_{\mathrm{ox}}}\left(\sqrt{4q\varepsilon_{\mathrm{SiC}}N_{A}\Phi_{F}}\right)$$

where *V*_FB_ is the flat band voltage, *Φ*_F_ is the bulk potential, *N*_ox_ is the trap charge density in the oxide, *C*_ox_ is the oxide capacitance, *q* is the elementary charge and *ε*_SiC_ is the dielectric constant of SiC.

In order to reach the MOSFET inversion condition, the bands must be bent to twice the bulk potential, *Φ*_F_ (the energy distance between the intrinsic level and the Fermi level in the bulk). Hence, due to its larger band gap, the surface electric field to reach the inversion in SiC is about two times higher than in Si [70], thus having an important impact on the scattering mechanisms that are strongly dependent on the surface electric field [65,71].

**Figure 7:**
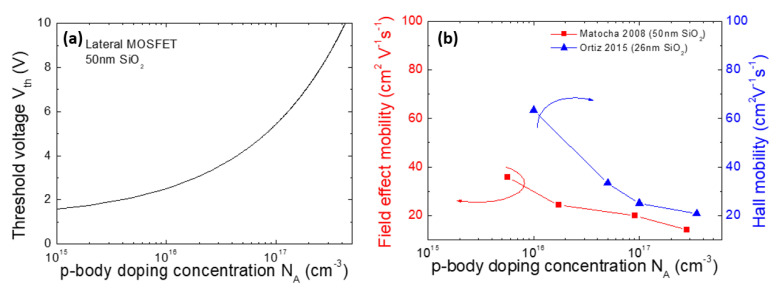
(**a**) Threshold voltage, *V*_th_, in lateral 4H-SiC MOSFET as a function of the p-body doping concentration, *N_A_*, calculated for an oxide thickness of 50 nm, considering a trapped charge density of 2 × 10^11^cm^−2^. (**b**) Field effect mobility and Hall mobility values determined in 4H-SiC MOSFET structures as a function of the p-body doping concentration. The data are taken from [70,74].

Figure 7a reports the threshold voltage, *V*_th_, of a lateral 4H-SiC MOSFET as a function of the active p-type doping concentration of the body, *N_A_*. The calculation has been carried out considering a SiO_2_ gate oxide thickness of 50 nm with a charge trap concentration of 2 × 10^11^ cm^−2^. As can be seen, the value of *V*_th_ increases by about 3 V when the p-type doping concentration, *N_A_*, varies from 1 to 3 × 10^17^ cm^−3^. This example further clarifies the importance of controlling the active doping level in the MOSFET body region. In addition, in order to predict and simulate the MOSFET electrical behavior, also the shape of the active doping concentration profile must be precisely known, since deviation from the nominal implanted “box-like” profile can occur after post-implantation annealing. For this purpose, depth-resolved techniques must be used. As an example, Giannazzo et al. [72] determined with nanometer resolution the acceptor depth profile in Al-implanted 4H-SiC regions by means of scanning capacitance microscopy (SCM) and scanning spreading resistance microscopy (SSRM), subjected to a post-implantation annealing at 1650 °C. Furthermore, two-dimensional SCM imaging allowed to visualize with a high spatial resolution the channel region of large-area 4H-SiC power MOSFETs and to estimate the homogeneity of the channel length over the whole device perimeter, clarifying the impact of the implantation doping parameters on the threshold voltage, *V*_th_, and the on-resistance, R_ON_, of the device [73].

The impact of the p-type doping concentration of the body region on the MOSFET mobility is shown in Figure 7b. In particular, the figure reports the behavior of the field effect mobility (determined in lateral MOSFETs) [70] and of the Hall mobility (determined by Hall bars) [74] for different values of the p-type doping. As can be seen, the mobility decreases with increasing doping concentration, which is due to the increase of the electric field in the channel region. As a consequence, a trade-off between the channel mobility and the threshold voltage is observed in 4H-SiC MOSFETs [68,74].

### 5.2. “Counter Doping” of 4H-SiC MOSFET Channel

One of the critical issues that has delayed the development of low-resistance 4H-SiC power MOSFETs has been the low inversion channel mobility. Such a limitation was mainly due to the poor electronic quality of the SiO_2_/SiC interface, which is characterized by a large density of interface states if compared with the SiO_2_/Si interface.

One of the first attempts to improve the channel mobility of 4H-SiC MOSFETs with a local doping method was the selective n-type implantation of the body region, proposed by Ueno et al. [75]. In particular, near-surface nitrogen implantation at a dose of 2 × 10^12^ cm^−2^ enabled an improvement of the field effect mobility in lateral MOSFETs up to 38 cm^2^V^−1^s^−1^. This nitrogen implantation process was named “counter doping”, because the electrical activation of the implanted nitrogen supplied donors that partially compensate the p-type doping of the channel region [75]. Almost ten years later, Moscatelli et al. [76] presented a similar study based on nitrogen implantation of lateral MOSFETs, achieving a field effect mobility of 21.9 cm^2^V^−1^s^−1^ at room temperature. However, the control of ion implantation-induced damage and of the thickness of the modified region were the main limitations of this method.

For that reason, in order to face the mobility problems in SiC MOSFETs, post-oxidation or post-deposition annealing processes in nitrogen-rich atmospheres (NO or N_2_O) were introduced more than two decades ago, and are nowadays the most widely used methods in both R&D and commercial devices [77]. Interestingly, a doping effect has also been demonstrated in nitridated SiO_2_/SiC interfaces. In particular, first, Swanson et al. [78] using SSRM and then Fiorenza et al. [79] using SCM demonstrated a “counter doping” effect of these nitridation processes, i.e., the nitrogen atoms incorporated in the near-surface region of the MOSFET channel are electrically active, and behave as donors, increasing the device channel conductivity. SCM was also employed to probe the effect of a P-implantation before the gate oxide deposition and N_2_O annealing on the lateral homogeneity of active donors at the SiO_2_/4H-SiC interface introduced by the nitridation process [80].

More recently, values of the field effect mobility larger than 100 cm^2^V^−1^s^−1^ have been obtained by using an antimony (Sb)-doped channel, created by ion implantation, in combination with post-oxidation annealing in NO [81].

Finally, Noguchi et al. [82] demonstrated a local doping of the 4H-SiC MOSFET channel by sulfur (S) ion implantation followed by the same activation annealing of the source/drain and p-body regions. In particular, different to conventional counter doping shallow donors, S introduces deep-level donors in the channel region, which enable to provide a low channel resistance while maintaining a high threshold voltage [82].

### 5.3. High-Dose n-Type and p-Type Doping for Ohmic Contacts in SiC Devices

As discussed in Section 3, in the case of high-dose implanted regions, the electrical activation of the dopants is typically more critical. Hence, the fabrication of Ohmic contacts on these implanted regions is strongly affected by the properties of these layers.

Several works investigated the properties of Al-implanted 4H-SiC layers, in many cases employing Hall measurements [38,56,83,84,85]. However, due the large variety of experimental conditions reported in the literature (implanted dose, annealing temperature, annealing time, etc.), even now this topic remains an object of scientific discussion.

**Figure 8:**
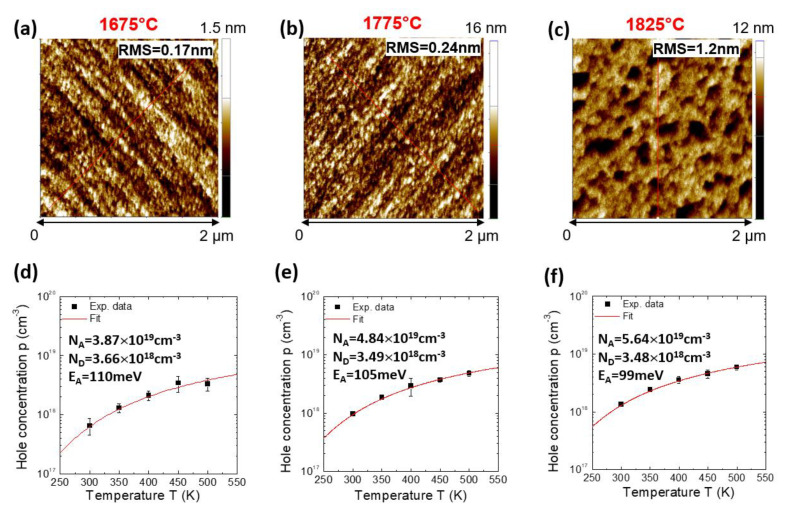
(**Top**) Surface morphology determined by AFM of high-dose p-type Al-implanted 4H-SiC after post-implantation annealing at (**a**) 1675 °C, (**b**) 1775 °C and (**c**) 1825 °C. (**Bottom**) Temperature dependence of the hole concentration for high-dose p-type Al-implanted 4H-SiC after annealing at (**d**) 1675 °C, (**e**) 1775 °C and (**f**) 1825 °C. The continuous lines are the fits of the data obtained using the neutrality equation. Adapted with permission from [86]. Copyright © 2021 Elsevier Ltd.

Recently, we monitored the effect of high-temperature (1675–1825 °C) post-implantation annealing on the electrical properties of p-type Al-implanted 4H-SiC, irradiated with energies in the range 30–200 keV and at a high dose (10^20^ at/cm^3^) [86]. In particular, the morphological AFM analyses of the annealed samples (see Figure 8a–c) revealed a significant increase of the surface roughness (RMS = 1.2 nm) only after annealing at 1825 °C. In this study, Van der Pauw and Hall Effect measurements have been used to estimate relevant parameters, such as the electrically active fraction of Al and the compensation. In particular, under the methodological point of view, to estimate the correct value of carrier concentration, the Hall scattering values, r_H_, should be carefully considered. Pensl et al. [87] first reported the temperature-dependent Hall scattering factor, r_H_, for Al-doping levels of about 1 × 10^18^ cm^−3^. More recently, Asada et al. [88] derived the values of r_H_ in a wider range of Al-concentration in 4H-SiC epilayers (5.8 × 10^14^–7.1 × 10^18^ cm^−3^). Hence, assuming the most appropriate values of r_H_ reported in [88], the Hall measurements at room temperature of the above-described samples could be corrected, resulting in hole concentration values in the range 0.65–1.34 × 10^18^ cm^−3^ and mobility in the order of 21–27 cm^2^ V^−1^ s^−1^ [86].

In general, for a p-type-doped 4H-SiC semiconductor with a doping density, *N_A_*, compensated with a donor’s density, *N_D_*, the temperature dependence of the hole density, *p*, can be determined from the neutrality equation [89]:

(3) $$\frac{p\left(p\;+\;N_{D}\right)\;-\;n_{i}^{2}}{N_{A}\;-\;N_{D}\;-\;p\;+\;n_{i}^{2}/p}\;=\;\frac{N_{V}}{g}exp\left(-\frac{E_{A}}{kT}\right)$$

where *n_i_* is the intrinsic carrier concentration of SiC, *N_V_* is the density of states in the valence band, *E_A_* is the ionization energy of the Al acceptor and *g* = 4 is the degeneracy factor of the ground level of the Al acceptor [87].

Since the intrinsic carrier concentration, *n_i_*, in SiC is extremely low, Equation (2) can be approximately rewritten as [90]:

(4) $$p\;\approx \;\frac{1}{2}\left[-N_{D}\;-\;x\;+\;\sqrt{{\left(N_{D}\;-\;x\right)}^{2}\;+\;4N_{A}x}\right]\;\mathrm{with}\;x\;=\;\frac{N_{V}}{g}exp\left(-\frac{EA}{kT}\right)$$

Hence, fitting the experimental temperature dependence of the hole density, *p* (Figure 8d,e), with the neutrality Equation (4) allowed estimating a fraction of active p-type Al-dopant increasing with the annealing temperature, i.e., 39% (1675 °C), 48% (1775 °C) and 56% (1875 °C), and a compensation decreasing from 9.4% to 6.2% in the same annealing conditions. Moreover, from the same analyses, it was found that the activation energy of the Al-implanted dopant species decreased with increasing the acceptor dopant concentration, from 110 meV (1675 °C) down to 99 meV (1875 °C).

**Figure 9:**
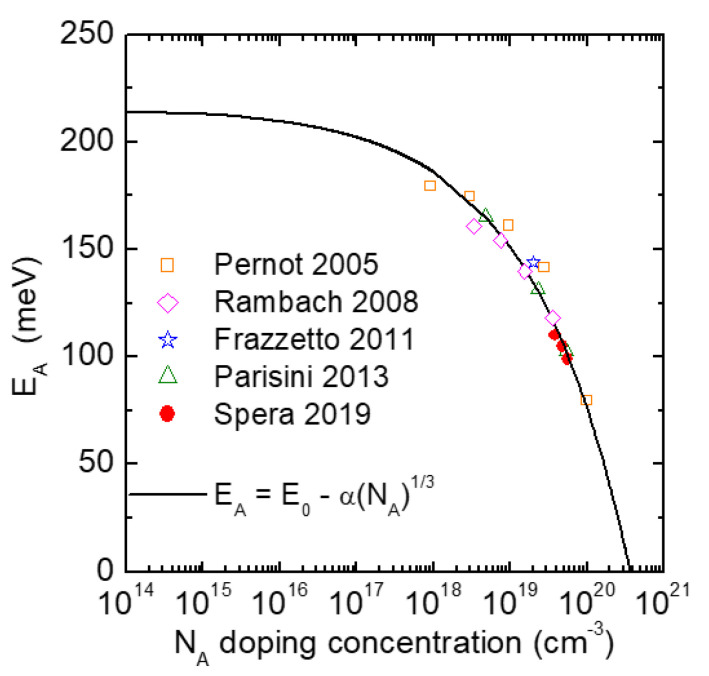
Ionization energy of the acceptors (*E_A_*) in 4H-SiC as a function of the p-type doping concentration (*N_A_*) determined by Hall measurements. The data are from [56,61,83,84,86] and refer to high doping levels (>10^18^ cm^−3^). The solid line corresponds to a fit with the Equation (1), with *E*_0_ = 216 meV and α = 3 × 10^−5^ meV cm.

Figure 9 reports a plot of the ionization energy, *E_A_*, as a function of the electrically active acceptor concentration, *N_A_*, found in this experiment [86], together with other literature results [56,61,83,84] on Al-implanted 4H-SiC at high doses. In particular, these data could be fitted with the theoretical behavior expressed in Equation (1), parametrized with an ionization energy *E*_0_ = 216 meV and a coefficient α = 3 × 10^−5^ meV cm.

The formation of Ohmic contacts to p-type 4H-SiC is a crucial issue, because of the high ionization energy of the acceptors and the difficulty to find metals with suitable work-function enabling to have a low Schottky barrier height [91]. Hence, while the typical metallization used for n-type materials, i.e., nickel silicide (Ni_2_Si), can also form Ohmic contacts onto heavily doped p-type 4H-SiC [92], Ti- and Al-based metallization schemes are preferred as they result in a lower barrier height and specific contact resistance [61,93,94,95,96]. In particular, recently, Ti/Al/Ni metal stacks annealed at 950 °C have been used to form good Ohmic contacts to p-type 4H-SiC [96].

Hence, this Ti/Al/Ni metallization scheme has been tested on the p-type implanted layers annealed under different conditions [97].

**Figure 10:**
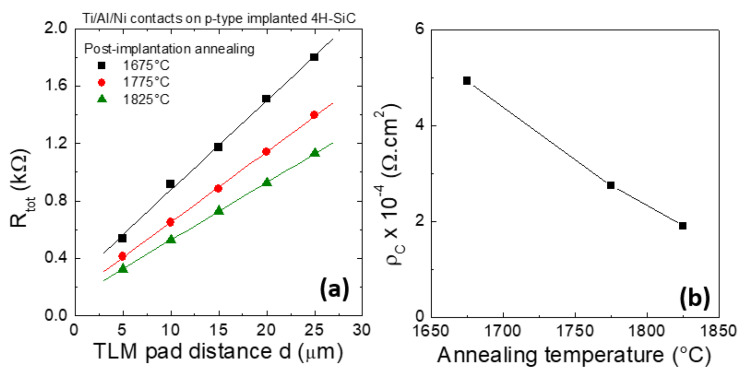
(**a**) Plots of the total resistance, R_TOT_, as a function of the TLM pad distance, d, used for the extraction of sheet resistance and specific contact resistance, for Ti/Al/Ni contacts on p-type Al-implanted 4H-SiC subjected to post-implantation annealing at 1675, 1775 and 1825 °C. (**b**) Specific contact resistance, *ρ*_C_, of the Ti/Al/Ni contacts as a function of the post-implantation annealing temperature. The figure is adapted from [97]. Copyright © 2021 by the authors; licensee MDPI, Basel, Switzerland. Reference [97] is an open-access article distributed under the terms and conditions of the Creative Commons Attribution License (http://creativecommons.org/licenses/by/4.0/).

Figure 10a reports the values of the total resistance, R_TOT_, as a function of the pad distances, d, in Transmission Line Model (TLM) structures used to evaluate the electrical properties of the contacts and implanted layers. As can be seen, the total resistance and the slope of the straight lines decreases with increasing the post-implantation annealing temperature, due to the decrease of the resistivity of the p-type layer. Moreover, the values of the specific contact resistance *ρ*_c_, determined by TLM analysis and reported in Figure 10b, decreased from 5.2 × 10^−4^ Ω cm^2^ (1675 °C) down to 2.6 × 10^−4^ Ω cm^2^ (1775 °C) and 2.0 × 10^−4^ Ω cm^2^ (1825 °C).

These results can be useful to set the optimal processing conditions for Ohmic contacts on the p-type regions in JBS and MOSFETs on 4H-SiC.

While in the case of Al-implanted 4H-SiC layers, the ionization energy and the electrically active fraction of the p-type Al-dopant are evaluated from the temperature dependence of the hole density applying the neutrality equation, for P-implanted n-type 4H-SiC layers, the neutrality equation has been applied to determine the electrical activation for doping levels up to 10^19^ cm^−3^ [39]. However, due to the lower ionization energy of the dopant, higher P-concentrations typically produce a degenerate semiconductor [31,32,33,98,99], for which the best way to evaluate the electrical activation is to determine the ratio of the electron concentration from Hall measurements with respect to the overall P-implanted dose, e.g., determined by SIMS.

In this context, we have recently elucidated the active dopant profiling and the Ohmic contact behavior in highly P-doped (~1 × 10^20^ cm^−3^) 4H-SiC implanted layers, by combining the integral carrier density measurement determined by Hall effect and scanning capacitance microscopy (SCM) carrier depth profiles [100]. In particular, Al-doped (~1 × 10^17^ cm^−3^) p-type 4H-SiC layers were implanted at 400 °C with P^+^-ions at multiple energies (30–200 keV) and doses (in the range 7.5 × 10^13^–5 × 10^14^ cm^−2^), in order to obtain a peak concentration in the order of 10^20^ cm^−3^. The implanted layers were subjected to post-implantation annealing at 1675 °C in Ar atmosphere for electrical activation.

**Figure 11:**
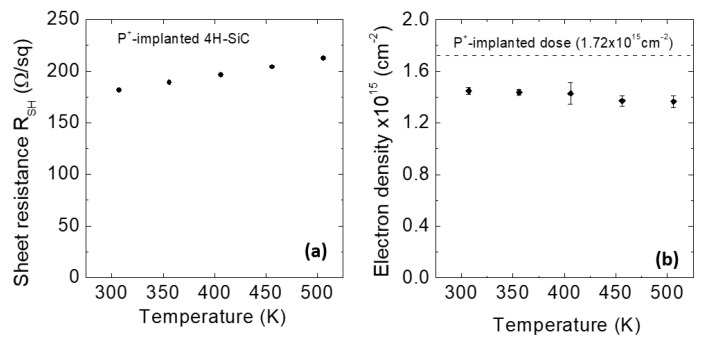
(**a**) Sheet resistance, R_SH_, and (**b**) electron density as a function of the temperature in the high-dose n-type P-implanted 4H-SiC samples. The dashed line represents the total implanted dose as determined by SIMS. Adapted with permission from [100]. Copyright 2020 © AIP Publishing.

Figure 11 reports the temperature dependence of the sheet resistance, R_SH_ (a), and of the electron density (b), of the P-implanted 4H-SiC, determined by Van der Pauw and Hall effect measurements, respectively. As can be seen, while R_SH_ only slightly increases from 181 to 212 Ohm/sq with increasing the measurement temperature (Figure 11a), the electron density (n = 1.44 × 10^15^ cm^−2^) is nearly independent of the temperature (Figure 11b), as expected for a degenerate semiconductor. Under these doping conditions, the donor energy level broadens and merges with the conduction band of 4H-SiC, thus allowing to assume that most of the P-donors are ionized.

Hence, the ratio between the electron density determined from Hall measurements and the P-dose given by SIMS profiles allowed for estimating an electrical activation of more than 80% of the implanted P^+^-ions.

**Figure 12:**
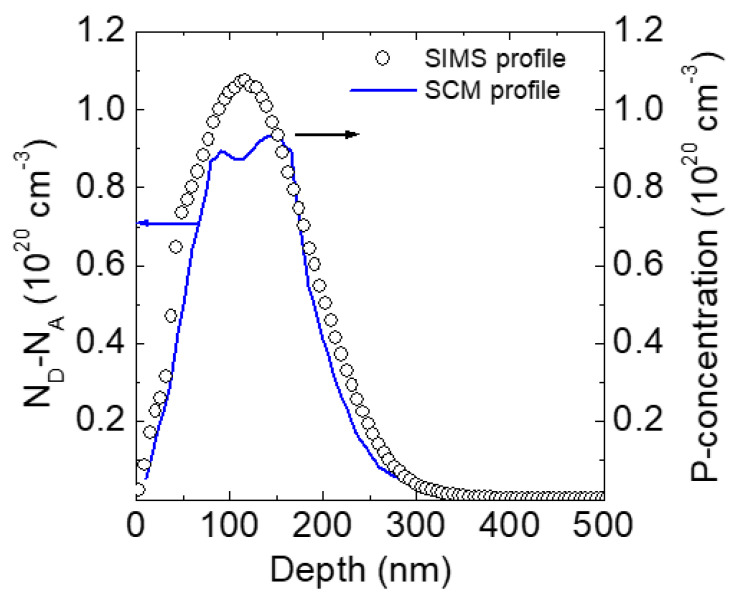
(Left scale, blue line) Electrically active dopant profile in high-dose P-implanted 4H-SiC, obtained by SCM normalized assuming the electron density determined by Hall measurements. (Right scale, black circles) Chemical P-concentration profile obtained by SIMS. Reprinted with permission from [100]. Copyright 2020 © AIP Publishing.

Figure 12 shows the electrically active P-profiles determined by SCM analysis profiles, using the Hall measurements to normalize the total charge density. For comparison, the SIMS profile is shown in the same plot. Notably, the SCM profile is almost overlapped with the SIMS profile both in the surface and in the tail of the P implant, whilst it exhibits a nearly flat region ~ (8.5–9) × 10^19^ cm^−3^, from 70 to 160 nm in depth. The lower electrically active concentration in this region can be attributed to the presence of a high density of implantation-induced structural defects [101], which lead to an incomplete activation of the implanted P-dopant in this region.

Achieving a high electrical activation is a key technological aspect for the fabrication of low-resistance Ohmic contacts on the n-type implanted 4H-SiC, e.g., in the source/drain regions of a MOSFET. In particular, nickel silicide Ohmic contacts fabricated on this n-type implanted layer by deposition of 100 nm Ni film and annealing at 950 °C exhibited a specific contact resistance, *ρ*_c_ = 5.4 × 10^−6^ Ωcm^2^ [100]. In this context, the knowledge of the depth distribution of the electrically active dopant in the implanted region, as that reported in Figure 12, and its value at the interface with the metal contact (e.g., a nickel silicide layer), is important to optimize the properties of Ohmic contacts and, ultimately, to minimize the specific on-resistance of the devices.

## 6. Non-Conventional Annealing and Doping Methods

While “hot implantation” followed by high-temperature annealing is now a well-consolidated method to achieve selective doping in SiC devices, in the last decade, alternative approaches for activation annealing and selective doping of the material have been proposed. In fact, as discussed in the previous sections, there are several issues affecting the conventional post-implantation annealing process, such as the difficulty to reach fast heating/cooling rates, the detrimental impact on the surface morphology, the poor electrical activation of some species, the difficulty of crystal damage recovery, etc.

Hence, in the next subsections, some of the interesting achievements obtained in SiC by non-conventional annealing and doping methods are briefly discussed.

### 6.1. Laser, Microwave and Plasma Annealing Technniques

Selective ion-implantation doping of SiC is a process that involves the generation of considerable amounts of defects, which can only partially be recovered by the conventional post-implantation annealing processes. In addition, the electrical activation is limited by the solid solubility of the implanted dopant species, and its kinetics is characterized by a transient behavior, i.e., the activation decreases rapidly with the time at a fixed temperature [51]. Hence, non-conventional rapid annealing processes can be a viable route to achieve the electrical activation of the dopant without incurring the aforementioned limitations. Laser annealing is a technique enabling very fast heating and cooling ramps and higher temperatures than the conventional furnace annealing. Today, laser annealing is mainly adopted for the formation of nickel silicide Ohmic contacts on the backside of 4H-SiC devices [102,103]. However, this technique can in principle be suitable to anneal implanted 4H-SiC layers as an alternative to the conventional furnace annealing, also providing the possibility to perform annealing processes in localized regions of the wafer.

Several attempts at activation of ion-implanted dopant in SiC by laser annealing have been reported in the last decades, typically using pulsed excimer lasers [104,105,106,107,108]. However, common to these works was the difficulty to recover the crystalline structure of the material. Recently, Calabretta et al. [109] achieved an almost stress-free crystalline lattice with respect to thermally annealed samples in P- and Al-implanted 4H-SiC samples subjected to multiple XeCl (308 nm) laser pulses of 30 ns duration.

Ultra-fast microwave annealing has been proposed to improve the electrical activation of dopant in SiC, due to the excellent selective absorption of microwave energy by high-dose implanted regions in the material [110]. In this method, microwaves are selectively absorbed by the SiC sample encased in a microwave transparent material, thus allowing a very fast heating and cooling of the material without heating the surrounding zone [110].

As an example, microwave annealing of ion-implanted SiC can provide ultra-fast ramp rates (>1000 °C/s) for annealing temperatures up to 2100 °C. This technique has been used by Nipoti et al. [111] for the electrical activation of high-dose (5 × 10^19^–8 × 10^20^ cm^−3^) p-type, Al-implanted, semi-insulating 4H-SiC, at annealing temperatures of 2000–2100 °C for 30 s. An electrical activation of 70% of the implanted Al (corresponding to a resistivity of 2 × 10^−2^ Ωcm) was achieved for the highest implanted dose of 8 × 10^20^ cm^−3^, at the annealing temperature of 2100 °C.

**Figure 13:**
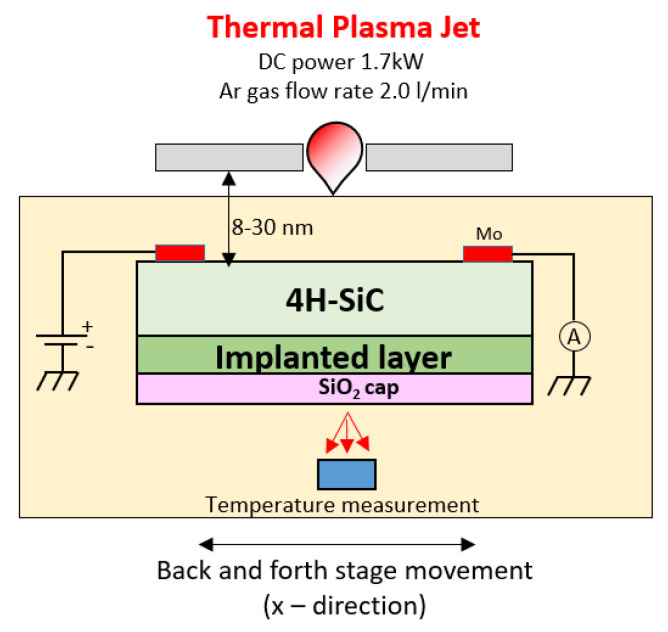
Schematic set-up of the thermal plasma jet (TPJ) annealing technique to achieve activation doping of ion-implanted SiC, proposed in [113,114].

Another alternative annealing technique is the so-called thermal plasma jet (TPJ). A TPJ is formed by blowing out an arc-discharge plasma through a very narrow nozzle of a few millimeters in diameter. The TPJ represents a non-conventional heat source that can reach a high-power density of several tens of kW/cm^2^ at atmospheric pressure [112]. The SiC sample is mounted on a motorized stage placed close to the TPJ nozzle, which enables the irradiation of the whole surface by an appropriate linear movement, while the temperature is measured by a radiation thermometer from the sample backside. The SiC wafers are preheated at temperatures in the range 800–1200 °C by a Joule heating system, while the rapid TPJ annealing is carried out in air. A SiO_2_ cap is deposited on the SiC implanted region in order to prevent oxidation during TPJ annealing. A schematic of the set-up used for the TPJ annealing is depicted in Figure 13. This method can enable a precise control of the heating and cooling rates in the millisecond range. Maruyama et al. [113] demonstrated the possibility to reach extremely high temperatures (>1800 °C) in a very short time (2.4 s). In this way, an efficient electrical activation was achieved in high-dose (>10^20^ cm^−3^) Al- and P-implanted samples [113,114].

As a conclusion of this section, in spite of some encouraging results reported in the aforementioned papers, the adoption of these new annealing techniques for electrical activation of ion-implanted dopant species in the fabrication of 4H-SiC devices seems to be not yet envisaged in the short term by the devices’ manufacturers.

### 6.2. Laser Irradiation Doping of SiC Immersed in a Liquid Containing Dopant Species

The use of laser irradiation to introduce dopants in SiC was originally proposed by Russell and Ramirez [115], who carried out boron doping of SiC by irradiating the material with a KrF excimer laser in diborane gas. In this way, a boron concentration of 10^18^ cm^−3^ could be incorporated in 4H-SiC.

Later, Tian et al. [116] performed n-type and p-type doping of SiC in nitrogen or trimethylaluminum (TMA) gas using an ArF excimer laser, obtaining an incorporation of dopant species at a concentration of 10^20^ cm^−3^.

Although these methods were proposed to create a shallow junction in SiC devices, they did not find any practical application in the following years.

**Figure 14:**
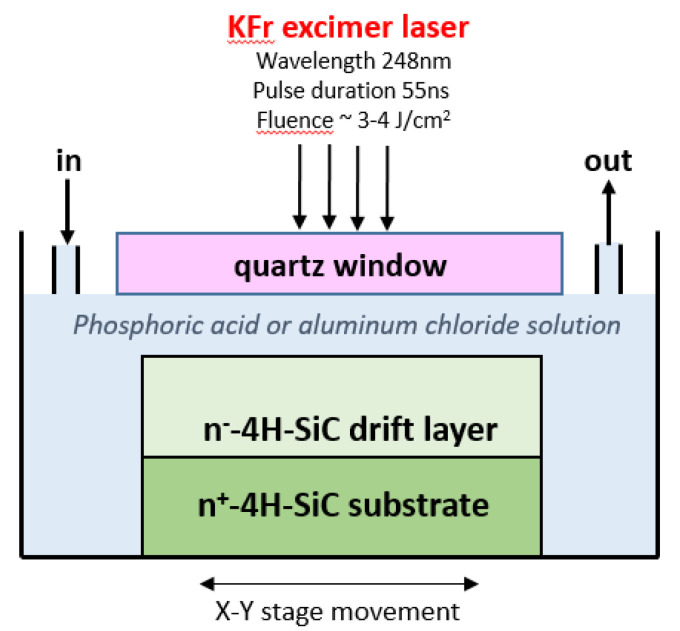
Schematic set-up of the excimer laser irradiation doping of SiC immersed in a liquid containing dopant species (phosphoric acid for n-type, aluminum chloride solution for p-type doping), proposed in [117,118].

More recently, Nishi et al. [117] investigated n-type phosphorous doping of 4H-SiC by irradiating with a KrF excimer laser, through a glass window, the material immersed in a phosphoric acid solution. The schematic set-up of this laser irradiation doping technique is shown in Figure 14. In particular, in this way, it was possible to achieve a phosphorous concentration up to 10^20^ cm^−3^ near the surface and the doping concentration could be tailored by changing the number of laser pulses. The same group succeeded in achieving aluminum doping of 4H-SiC by excimer laser irradiation of the material immersed in aluminum chloride solution [118]. In particular, Al was introduced near the surface with a concentration of 10^20^ cm^−3^, and an electrical activation of about 1% was achieved, which in turn allowed the demonstration of p-n junctions with several decades of on/off ratio.

However, using this method, the doping depth of P or Al is limited to about 30–40 nm, even with increasing the laser fluence or the shots number. Hence, it can be applied as a sub-contact doping technique to improve the Ohmic contact properties.

### 6.3. Other p-Type Doping Methods Based on Al Melting

As specified in the previous subsection, laser irradiation of SiC immersed in a liquid allowed achieving doping of the near-surface region of the material (30–40 nm). To overcome this limitation, Ikeda et al. [119] obtained Al doping of 4H-SiC with a high surface concentration and deep depth profile by irradiating single-pulse (4 J/cm^2^) excimer laser to Al films (60–800 nm) deposited on the SiC surface. In particular, high-temperature molten Al is produced behind the laser-generated high-density Al plasma, and Al is diffused from the molten Al into 4H-SiC. After irradiation, Al is removed by wet etch. In this way, the Al doping depth could be notably increased, up to ~200 nm, and the p-n junction diode fabricated by the doping with the molten Al showed an on/off ratio over 10 orders of magnitude.

Park et al. [120] proposed a method to form “shallow” p-doping on a 4H-SiC surface by depositing a thin Al layer (~5 nm) and then thermally annealing it at 1000 °C for 10 min. Secondary ion mass spectrometry (SIMS) analysis of the annealed Al/SiC sample revealed an Al concentration higher than 10^17^ cm^−3^ over a depth of about 250 nm. Current-voltage and capacitance-voltage measurements on SBDs fabricated on an n-type SiC epilayer showed that the shallow Al doping increases the built-in potential of the junction and the barrier height by 0.51 and 0.26 eV, respectively. This effect can be attributed to a partial dopant activation (activation ratio 2%). Then, this “shallow doping” method could be used to fabricate junction terminations, which reduced the leakage current and improved the breakdown voltage in SBDs.

**Figure 15:**
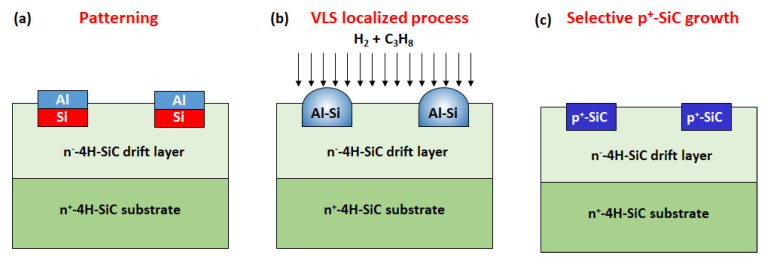
Schematic representation of the local p-type doping of SiC by the VLS process: (**a**) an Al-Si stack is deposited on the SiC substrate and defined by photolithography and etching, (**b**) the sample is heated above the melting of the Al-Si, and a propane flow is added in order to obtain the VLS growth, (**c**) the formation of p^+^-SiC locally occurs below the Al-Si liquid. After this VLS sequence, the unreacted Al-Si alloy is removed using acid solutions. More details on this techniques are reported in [121,122,123].

Finally, Ferro et al. [121] developed an alternative approach for SiC growth, called VLS (vapor–liquid–solid), where a Si-based melt is fed by an alkane (e.g., C_3_H_8_) in order to grow the SiC layer on a seed. The addition of specific metals, which can be incorporated into the SiC lattice during growth, can confer particular properties to a grown layer, such as heavy p-type doping in the case of Al. A schematic of this local doping method is reported in Figure 15.

Indeed, by using this approach, a localized heavily p-type SiC epitaxy could be achieved and employed for the fabrication of p-n junctions [122,123].

## 7. Conclusions

This paper provided a short overview of the selective doping techniques for silicon carbide (4H-SiC) power devices technology. In particular, ion implantation is the method of choice for selective doping of SiC and nowadays represents the only technique used by the industrial device manufacturers.

Phosphorous and aluminum are the most common n-type and p-type dopant species for 4H-SiC SiC devices. They are implanted at 300–500 °C (hot implantation) and electrically activated by post-implantation annealing processes at high temperatures, in the range 1500–1800 °C. During this process, the sample is encapsulated by a protective carbon-based capping layer, to avoid SiC surface degradation. The complete activation of the dopants can be typically achieved for low implanted doses, while only partial activation is reached when the implanted dose exceeds 10^15^ cm^−2^. Moreover, independent of the implanted dose, Al-incomplete ionization at room temperature is a typical concern of p-type doping, due to the high activation energy level of this acceptor. The control of the electrical activation of the dopant species and the accurate knowledge of the depth profiles of the active impurities are very important, due to the relevant implications in power devices. In fact, many device parameters in diodes and transistors, e.g., specific contact resistance, threshold voltage, channel mobility, etc., are critically influenced by the quality of the implanted layer and the active doping level. In spite of the great progress achieved in the last three decades in this field, ion-implantation doping of SiC remains a subject of intensive investigation by the scientific community. Some recent results on heavily doped P- and Al-implanted 4H-SiC layers have been reported in this paper, discussing the impact on the specific contact resistance of Ohmic contacts fabricated on these layers.

Although ion implantation is a consolidated method to selectively dope 4H-SiC during device fabrication, the reduction of ion beam-induced damage and the improvement of the electrical activation in heavily doped layers remain important challenges. In order to overcome these limitations, recently, new annealing methods and selective doping techniques have been explored (e.g., based on laser, microwaves, plasma jet, etc.), to further improve the electrical activation of the dopant by fast heating rates. While these attempts provided interesting results and simple p-n junctions have been demonstrated, the control of the junction depth remains a great concern. Hence, the application of these methods in large-scale device production is not yet foreseen in the short term.

## Figures and Tables

**Table 1:** Ionization energies and solubility limits of the major dopants in 4H-SiC. The values of the ionization energies for N, P and Al refer to the hexagonal (h) and cubic (k) sites [1,3,4].

Dopant	Ionization Energy (meV)	Solubility Limit (cm^−3^)
N	61(h); 126 (k)	2 × 10^20^
P	60 (h); 120 (k)	1 × 10^21^
Al	198 (h); 201 (k)	1 × 10^21^
B (shallow)	280–300	2 × 10^19^

**Table 2:** Typical doping levels and implantation depths of main implanted junctions found in SiC devices.

Implanted Region	Doping Level (cm^−3^)	Implantation Depth (μm)
Source/Drain	10^19^–10^20^	0.2–0.3
MOSFET p-body	10^17^–10^18^	0.4–0.8
p^+^-contacts	10^19^–10^20^	0.2–0.3
Edge termination	10^16^–10^17^	0.4–0.8

## Data Availability

The data that support the findings of this study are available from the corresponding author upon reasonable request.
